# Shared attention for action selection and action monitoring in goal-directed reaching

**DOI:** 10.1007/s00426-018-1064-x

**Published:** 2018-08-10

**Authors:** Aoife Mahon, Solveiga Bendžiūtė, Constanze Hesse, Amelia R. Hunt

**Affiliations:** 1 School of Psychology, University of Aberdeen, Aberdeen, UK; 2 Institute for Health Research, University of Bedfordshire, Luton, LU2 8LE UK

## Abstract

Dual-task studies have shown higher sensitivity for stimuli presented at the targets of upcoming actions. We examined whether attention is directed to action targets for the purpose of action selection, or if attention is directed to these locations because they are expected to provide feedback about movement outcomes. In our experiment, endpoint accuracy feedback was spatially separated from the action targets to determine whether attention would be allocated to (a) the action targets, (b) the expected source of feedback, or (c) to both locations. Participants reached towards a location indicated by an arrow while identifying a discrimination target that could appear in any one of eight possible locations. Discrimination target accuracy was used as a measure of attention allocation. Participants were unable to see their hand during reaching and were provided with a small monetary reward for each accurate movement. Discrimination target accuracy was best at action targets but was also enhanced at the spatially separated feedback locations. Separating feedback from the reaching targets did not diminish discrimination accuracy at the movement targets but did result in delayed movement initiation and reduced reaching accuracy, relative to when feedback was presented at the reaching target. The results suggest attention is required for both action planning and monitoring movement outcomes. Dividing attention between these functions negatively impacts action performance.

**Electronic supplementary material:**

The online version of this article (10.1007/s00426-018-1064-x) contains supplementary material, which is available to authorized users.

## Introduction

Although there is still some debate about the details, it is generally agreed that attention is pivotal in the acquisition, development and retention of new skills, as well as the planning and execution of simpler actions such as saccades and hand movements (Deubel & Schneider, 1996; Schneider & Deubel, 2002; Baldauf, Wolf, & Deubel, 2006). Visual attention is widely considered to serve two main functions: selection-for-perception and selection-for-action (Allport, 1987). Broadly defined, selection-for-perception refers to the extraction of information relevant to functions such as object identification and scene understanding (Posner, 1980; Treisman & Gelade, 1980). Selection-for-action, again broadly defined, refers to the extraction of information that is relevant for planning goal-directed actions (Allport, 1987; Neumann, 1987). During the planning and programming of an action, numerous studies have found that attention is allocated to relevant movement goal locations, leading to faster and more accurate visual discrimination at these locations (e.g., Baldauf, Wolf, & Deubel, 2006; Baldauf & Deubel, 2008a, b). This has been interpreted as evidence that selecting a target for action also facilitates perceptual processing of the target, suggesting these two functions of attention are linked.

The strong coupling between perceptual facilitation and action has commonly been demonstrated using dual-task paradigms. In these studies, participants are required to execute a goal-directed movement while simultaneously identifying a discrimination target at varying locations. Discrimination target identification is used as a measure of attention allocation. Presenting the discrimination target before movement initiation at the same location as the movement target enhances identification compared to all other locations (Deubel & Schneider, 1996; Baldauf, Wolf, & Deubel, 2006; Baldauf & Deubel, 2008a, b; Jonikaitis & Deubel, 2011). When participants are required to execute more than one pointing movement, discrimination target identification accuracy was found to be enhanced at all relevant movement targets but not at the locations between them (Baldauf, Wolf, & Deubel, 2006), suggesting multiple selective foci of attention and not a broad spotlight effect of attention.

Correct discrimination target identification requires selection-for-perception, while movement planning and control requires selection-for-action. The findings of the dual-task studies suggest that these two attentional functions are intertwined (Schneider, 1995; Deubel & Schneider, 1996). Although this coupling is generally considered to be obligatory within these studies, it is unclear why it exists. One proposal is that object recognition and action selection share a single, common attention resource (Schneider, 1995). Another proposal is that shifts of attention are equivalent to motor preparation (premotor theory, Rizzolati, Riggio, Dascola, & Umilta, 1987). In the current study, we suggest a third option: attention is allocated to expected sources of task-relevant information about action outcome. Comparing the visual effect of an action to its desired or expected effect is a perceptual task that is likely to require selective attention, so it seems likely that attention would be allocated to this source of information, perhaps even before the action begins.

Note that not all studies have shown enhanced perceptual performance at movement targets (e.g. Bonfiglioli, Duncan, Rorden & Kennett, 2002, Remington, 1980; Stelmach, Campsall & Herdman, 1997; Belopolsky & Theeuwes, 2009), suggesting that the coupling between action and attention may not be as obligatory as originally suggested. For example, Bonfiglioli et al. (2002) found no perceptual enhancement at the target of reaching movements when the hand and target were hidden from view. As their studies only included older participants (age range 58–69 years), these results could be interpreted as evidence for an age-related difference in perception and action coupling. However, Fischer (1997) also found no effect of motor preparation on perceptual performance in younger adults (mean age 21 years) during pointing. Similä and McIntosh (2015) argue that predictable hand movements can be pre-programmed, meaning they may be executed without concurrent target selection. More generally, experiments examining the link between attention and action need to provide explicit directional cues to participants to execute movements towards particular locations, to be able to measure attention at these locations before the movement begins. Movements that follow directions may engage attention-for-perception, but it is an open question whether free movements in more natural circumstances have the same attentional demands (Hunt, Reuther, Hilchey & Klein, 2018). If attention is usually, but not always, allocated to movement targets, attention cannot be required for action selection to occur. An alternative possibility is that attention is allocated strategically, to monitor movement outcomes.

There is some support for the idea that attention is selectively allocated to monitor movement outcome accuracy. For example, after making a movement error, individuals tend to be slower and more cautious on the subsequent trials (e.g., Dutilh et al., 2012; Kerns et al., 2004; Rabbitt, 1966). The change in post-error movement characteristics provides support for the existence of a performance monitoring system, where attention is needed to reduce movement errors in upcoming trials (e.g., Gehring, Goss, Coles, Meyer, & Donchin, 1993; Hester, Foxe, Molholm, Shpaner, & Garavan, 2005; Kearns et al., 2004; van Veen & Carter, 2005). More recently, Kunde Wirth and Janczyk (2018) suggested that feedback monitoring may be partially responsible for the psychological refractory period effect (Pashler & Johnston, 1989; Welford, 1952). The psychological refractory period effect, commonly reported in dual-task studies, refers to the slowed response exhibited towards a second stimulus (e.g., Telford, 1931). This effect is usually explained as a consequence of the overlapping processing of the first stimulus with processing of the second stimulus, with the period between encoding and action execution being capacity limited (Navon & Miller, 2002; Pashler, 1994; Tombu & Jolicoeur, 2003). In their study, Kunde and colleagues asked participants to perform two tasks with each task producing visual effects, i.e. feedback. Crucially, the visual feedback was either immediate or delayed. They found that delaying visual feedback from the first task delayed responding to the second task. It was suggested that delaying the feedback resulted in participants monitoring the first task for longer, thus causing a temporal overlap with the processing of the second task. In a follow-up study, Wirth, Janczyk and Kunde (2018) confirmed that attention was allocated to “effect monitoring” (i.e. monitoring the outcome/effect of actions), arguing that this monitoring is not only crucial for identifying and interpreting response–effects in our environment, but also for learning response–effect associations to begin with.

In skilled action learning, participants who allocate attention to monitor their movement outcomes usually perform better than those who attend to the movement itself (Keller, Lauber, Gottschalk & Taube, 2015; for a review see Gray, 2011). Generally, in these studies participants are provided with augmented feedback and encouraged to use this information to improve their movement performance. Augmented feedback refers to extrinsic information about movement performance (such as a visual re-play of a movement) and is used for error correction and to guide and improve future actions (Lauber & Keller, 2014; Schmidt & Lee, 2011; Keller, Lauber, Gottschalk & Taube, 2015). Augmented feedback that provides more information than intrinsic task information alone has been found to accelerate skill acquisition, as it provides participants with information they can use to optimize their performance. Classic dual-task studies showing a coupling between attention and reaching (or pointing) movements are often performed open-looped with the hand obscured from sight. This can result in participants being provided with a combination of both task-intrinsic and extrinsic feedback. For example, in previous dual-task experiments (e.g. Baldauf, Wolf, & Deubel, 2006; Baldauf, & Deubel, 2008b), participants were required to point to cued targets, and to simultaneously identify a discrimination target, while their hand was hidden from view (visual stimuli were displayed on a mirror with the hand underneath). This eliminated visual feedback about the accuracy of the movement that would normally come from perceiving the finger’s location relative to the target at the end of the pointing action. Instead, participants were provided with augmented feedback: at the end of each trial, participants were shown a red dot that provided visual feedback about the final landing position of their pointing finger, and a tone provided feedback about their reaction time. By providing participants with additional feedback information in the same location as the action target, it is unclear whether attention was allocated for the purpose of movement target selection, or to monitor the provided feedback information.

The idea that attention is allocated to action targets for the purpose of monitoring action outcomes is a novel alternative to the proposal that attention is required for motor selection (VAM, Schneider, 1995) or that motor planning causes automatic shifts of attention (premotor theory, Rizzolati et al., 1987). Here we test this ‘attention-for-feedback’ hypothesis of attention allocation in action execution. In addition to using discrimination target accuracy to determine the locus of attention, we also examined movement characteristics in the current study. While studies examining the coupling of attention and action generally require participants to execute movements, how attention allocation impacts movement characteristics is not as widely reported. Baldauf et al. (2006) did measure whether discrimination target location affected reaction times and movement times but found no reliable effects. Hesse and Deubel (2011) showed that withdrawing attention from the grasping movement locations resulted in less efficient grasp preparation and execution (see also Hesse, Schenk, & Deubel, 2012; Similä & McIntosh, 2015). Separating selection-for-perception and selection-for-action in the above experiments resulted in dual-task costs. As selective attention has been suggested to be a limited resource (Kahnemann, 1973), the dual-task costs reported in these studies demonstrate these processing limitations and provide further evidence for these two functions sharing common attentional resources.

Another question we want to address in the current study is the extent to which enhanced discrimination performance at the goal of the action is due to the immediate selection of an action target, versus the general relevance of this location for upcoming actions, accumulated over the course of the experiment. Classic dual-task studies have not differentiated between locations that are possible movement targets in other trials, relative to locations that are never the target of a movement during an experiment. Discrimination accuracy may be enhanced only at locations that are currently selected as the target for an impending action. Alternatively, there may be an attentional hierarchy, with the immediately-targeted location being prioritized over potential movement-relevant locations, followed by locations that are never indicated as a target for movement.

Our primary aim in this experiment was to dissociate the location providing information about movement outcome from the movement target locations. This procedure allows a direct comparison between visual discrimination performance at the feedback locations and the action target locations. Separating the feedback locations from the movement targets has the consequence that no useful visual information about the movement outcome can be expected to arise from the movement target locations. If attention really is needed for action selection, we should see enhanced visual discrimination at the action target even under these conditions. We also examine the consequences of spatially shared vs. separated visual feedback on movement characteristics: if attention is necessary for action selection, then action planning and movement control should be negatively affected by the requirement to split attention with a spatially separated feedback location.

## Methods

### Participants

Sixteen undergraduate and postgraduate students of the University of Aberdeen, including two of the authors (A.M. & S.B.), age range 22–36 years, average age of 25.6 years (6 females), were paid to participate. In total, each participant completed 1280 experimental trials. Fourteen participants remained after two participants were excluded from analysis: one participant could not complete the perceptual task above chance in any of the tested conditions, and the second participant could not complete the reaching task within the specified accuracy requirements (see procedure for more details). All participants had normal or corrected-to-normal vision and were right-handed by self-report. Informed consent was obtained from all individual participants included in the study. This research was approved by the Psychology Research Ethics Committee, University of Aberdeen.

### Set-up

**Fig. 1:**
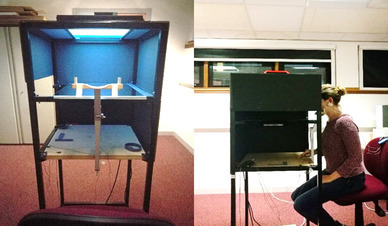
Photograph of the experimental apparatus

A photograph of the set-up can be seen in Fig. 1. The experiment was performed visually open-loop by use of a mirror set-up and conducted in a dimly illuminated room. A computer monitor (EIZO Foris FG2421, 23.5″, 100 Hz, 1920 × 1080 pixel) was secured into a metal frame with the screen facing downwards, reflecting stimuli onto a semi-transparent mirror (56 cm × 40 cm) positioned 34 cm below. The light of the computer monitor reflected off the mirror, resulting in light above the mirror and darkness underneath, such that participants were unable to see their hand. Participants were required to reach towards the relative stimulus positions on the table surface, located 34 cm underneath the mirror. Participants sat on a height-adjustable chair with their head on a chinrest. Their right index finger was placed at the starting position which was marked by a felt pad (5 mm in diameter) and centrally aligned in front of the chin rest. Fixation was controlled using a BlueGain electro-oculogram (EOG) amplifier (Cambridge Research Systems, Kent, England). Two electrodes were placed around the left eye, with one attached above the right edge of the left eyebrow and the other parallel with the pupil toward the left temple. An additional earth electrode was attached to the left earlobe. It was ensured at the start of each block that vertical and horizontal eye-movements were easily detectable. Eye-movements resulting in voltage changes of about 10–20 microVolts could be reliably identified and correspond to eye-movements of about 2° of visual angle (depending on skin condition, tiredness, etc. of participants, see Ross, Schenk, & Hesse, 2015 for similar procedure). Eye-movements were re-calibrated after every block for each participant.

Reaching movements were recorded using an infrared Optotrak 3020 motion tracker system (Northern Digital Incorporation, Waterloo, Ontario, Canada) at a sampling rate of 200 Hz. A single infrared light emitting diode (IRED) was placed on the tip of the right index finger. EOG and Optotrak were synchronized using an infrared signal transmitted to the EOG at the beginning and end of each trial. Data were monitored in real time by the experimenter. This allowed the experimenter to discard any trials in which an error had occurred, for example, when an eye-movement was made during a trial, or when participants executed early hand movements. These trials were returned to the trial list to be presented again, randomly ordered within the remaining trials of the experimental block (“recycled”). The experimental script was created and run using MatLab2012b with the Psychophysics Toolbox version 3.0.8 (Brainard, 1997) and Optotrak toolbox (Franz, 2004).

### Stimuli and procedure

**Fig. 2:**
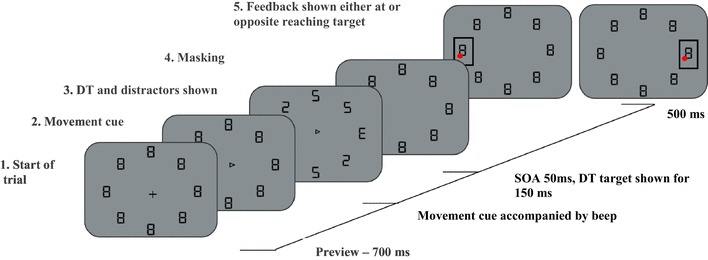
Illustration of a trial sequence. Feedback was shown either at the character opposite of the movement location (Different condition) as seen on the left of the above image, or at the movement location (Same condition) as seen on the right

Each trial began with a black central fixation cross, 5 mm × 5 mm, presented on a grey background, surrounded by a circular array of eight black pre-masked targets, with an imaginary radius of 5.0° from the fixation cross. All targets displayed resembled a digital ‘8’, with a horizontal width of 6 mm and a height of 10 mm. After a presentation time of 700 ms, the central fixation cross was replaced with a central movement cue, represented as a black arrow, 5 mm × 5 mm, which pointed to one of the surrounding targets. The movement cue indicated which target participants were required to reach towards and was simultaneously presented with an acoustical beep (1000 Hz for 100 ms). Movement targets were always presented on one of the four cardinal locations (i.e. “left”, “right”, “up” and “down”). The beep signalled that participants had to initiate their movement to the movement target. Participants were instructed to maintain central fixation throughout the trials. After a stimulus onset asynchrony (SOA) of 50 ms after the central movement cue was displayed, seven out of the eight pre-masked targets changed into distractors, shown as either a digital ‘2’ or ‘5’ (randomly determined), while one character changed into a critical discrimination target (DT) which resembled either a digital ‘3’ or ‘E’. For all locations, the discrimination target was equally often presented as either a ‘3’ or an ‘E’. Distractors and the critical discrimination target were presented for 150 ms, after which all eight targets changed back to the initial pre-mask character of a digital ‘8’, see Fig. 2. Participants were asked to verbally indicate if they had seen a ‘3’ or an ‘E’ at the end of each trial.

Each trial had a determined length of 2 s. Within this trial duration, the end of the movement and the landing position of the finger were determined. Final landing positions were deemed accurate if participants reached within ± 8 mm from the outer edges of the movement target, along the *x*- and *y*-axis. Outside of these parameters, the movement was deemed inaccurate. For each accurate movement, participants received a 3 pence monetary reward. No reward was provided for inaccurate trials. Immediately after completing their movement, participants received visual feedback regarding their movement accuracy. Feedback was either presented at the movement locations in one condition (*Same*) or, in a second condition, spatially separated at the location opposite the reaching target (*Different*), see Fig. 2. Feedback was presented by showing an accuracy box (in black lines) around the feedback target (8 mm × 8 mm from the target’s outer edges), and a red dot showing the actual final landing position of the finger at the end of the trial (see Fig. 2 for illustration). Feedback was displayed for 500 ms.

### Design

There were two main experimental manipulations, Feedback Location and DT Location. Feedback Location refers to whether the feedback was provided at the Same location as the reaching target or opposite at a Different location. This variable was blocked. DT Location was randomized within each block and fell into four categories:

1. *Movement*: The DT was shown at the reaching target. Movement location was one of the four cardinal locations (i.e. “left”, “right”, “up” and “down”).
2. *Separate Feedback*: The DT was shown at the feedback location (opposite the reaching target, see Fig. 2). This category was only possible when the feedback location was spatially separated from the movement location (i.e. Different condition).
3. *Movement Other*: The DT was shown at a location that was a potential movement location on a previous trial (i.e. the remaining three cardinal locations).
4. *Irrelevant*: The DT was shown in one of the inter-cardinal locations that were never reaching locations, i.e. movement irrelevant.

In total, each participant completed 1536 trials over 12 blocks (128 trials per block), including two practice blocks and ten experimental blocks. Participants completed three blocks per session across four sessions. Blocks lasted roughly 20 min. The feedback condition (Same and Different locations relative to the action target) was blocked and the blocks were counterbalanced and presented in ABAB and BABA design across participants. Each block of the Same condition consisted of 16 Movement trials, 48 Movement Other trials, and 64 Irrelevant trials. Each block of the Different condition consisted of 16 Movement trials, 16 Separate Feedback trials, 32 Movement Other trials, and 64 Irrelevant trials. Participants were paid £30 for completing all sessions, and an additional 3 pence for each ‘accurate’ trial they completed. Movement accuracy was only rewarded in experimental blocks resulting in a maximum possible additional reward of £38.40.

### Data analysis and rejection of trials

All EOG data were presented in real time on the experimenter’s display monitor and were checked online. All hand-movement data were checked online for missing data and later checked again offline. Trials in which participants did not maintain central fixation (as indicated by the EOG recording) reached towards the wrong target, or when the finger marker was not visible (as indicated by missing data in the trajectory) were discarded online and recycled. To ensure that the discrimination target was no longer present when the movement was initiated, all trials with a movement onset latency below 200 ms were excluded from further analysis, as 200 ms is equivalent to the 50-ms SOA and the 150-ms discrimination target presentation time. In total 103 (1.2%) Same and 94 (1%) Different trials were removed from further analysis due to reaction times being shorter than 200 ms. Trials in which participants did not finish the reach within the 2-s duration of the experimental trial were discarded online and recycled. The perceptual task was 2-AFC as two DT alternatives were presented (“E” or “3”). A non-parametric binomial test (test proportion = 0.5) was conducted to assess whether individual participants performed above chance in any of the experimental conditions. As noted above, one participant did not perform the perceptual task above chance and was removed from further analysis.

The IRED placed on the participants’ right index finger was used to determine hand position throughout the 2 s trial duration. In a first step, all hand movement data were filtered offline using a second-order Butterworth Filter with a low-pass cut-off frequency of 15 Hz. From the filtered positional data, we calculated the resultant velocity of the marker. Movement onset was defined as the moment the participants’ finger marker exceeded a velocity threshold of 0.10 m/s. The time between the onset of the movement cue and movement onset was defined as reaction time (RT). The end of movement was defined as the point at which the velocity of the finger marker dropped below a threshold of 0.05 m/s. Movement time (MT) was calculated as the time between movement onset and end of the movement. Endpoint accuracy (absolute error) was measured in 2D space (along the *x* and *y* dimension) and calculated as the Euclidean distance between the centre of the target and the participant’s final landing position as determined at the end of movement. Endpoint accuracy was determined in the *x* and *y* dimensions only, because all targets were presented on the table-plane (i.e. constant z-dimension). All statistical analysis was conducted using means.

Following similar previous studies (e.g., Baldauf, Wolf, & Deubel, 2006; Baldauf, & Deubel, 2008b), to investigate differences resulting from DT Location, statistical analyses included one-way repeated-measures analyses of variance (ANOVA) for each dependent variable listed above. A Greenhouse–Geisser correction was applied if the sphericity assumption was violated (Geisser & Greenhouse 1958). Unadjusted degrees of freedom and epsilon (ε) values are provided if Greenhouse–Geisser correction was applied. A significance level of α = 0.05 was used for all analyses. Bonferroni corrections were used for all post hoc comparisons. Participants took part in all experimental conditions. In all graphs, error bars represent within-subjects standard error (Cousineau 2005). In our descriptive results, we present within-subjects standard errors. Statistical analysis was conducted using the statistical software IBM SPSS Statistics 24.

## Results

### Discrimination target accuracy

**Fig. 3:**
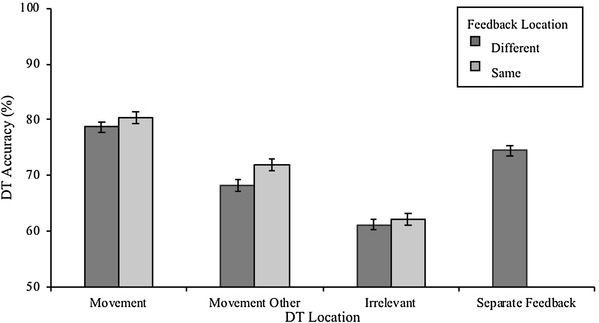
Mean discrimination target identification accuracy. DT Location refers to the location the discrimination target was presented. *Same* means the feedback was provided at the Movement Location and *Different *means feedback was provided opposite the Movement Location. Chance level corresponds to 50% correct. Error bars represent within-subjects standard errors

As can be seen from Fig. 3, DT accuracy was highest at the reaching targets and was not diminished even when feedback came from a different location. DT accuracy was also high at feedback locations, suggesting attention was allocated to both locations when they were spatially separated, with no apparent cost to overall perceptual accuracy at the movement locations.

The statistical significance of this pattern of results was confirmed using one-way repeated-measures ANOVAs comparing the effect of DT Location separately for Same and Different feedback locations (separate analyses were conducted because of the difference in the number of DT categories across the Same and Different feedback blocks). In Same feedback location blocks, there was a significant effect of DT Location, *F*(2, 26) = 57.3, *p* < 0.001, η_*p*_^2^ = 0.883. Post hoc comparisons showed that perceptual performance was enhanced at Movement locations compared to both Movement Other (*M*_diff_ = 8.4 ± 1.52%, *p* < 0.001), and Irrelevant locations (*M*_diff_ =18.3 ± 1.85%, *p* < 0.001). Furthermore, DT Accuracy at the Movement Other locations was enhanced compared to Irrelevant locations (*M*_diff_ = 9.9 ± 1.74%, *p* < 0.001).

In the repeated-measures ANOVA on the Different feedback location condition, DT location was a four-level factor: Movement, Movement Other, Irrelevant, and Separate Feedback locations. The main effect of DT Location was significant, *F*(3, 39) = 27.6, *p* < 0.001, η_*p*_^2^ = 0.864. Perceptual performance at the Movement locations was enhanced compared to Movement Other (*M*_diff_ = 10.5 ± 1.97%, *p* = 0.001) and Irrelevant locations (*M*_diff_ = 17.5 ± 2.14%, *p* < 0.001). DT Accuracy at Movement Other was also significantly enhanced compared to Irrelevant locations (*M*_diff_ = 7.0 ± 2.22%, *p* = 0.046). All possible movement locations, therefore, showed enhanced attention allocation compared to movement-irrelevant locations, with current reaching targets receiving the most attention. Most importantly, DT identification accuracy was also enhanced at the Separate Feedback locations (*M* = 74.5 ± 2.9%) in the Different blocks, which did not differ from the discrimination performance at the Movement locations (*M*_diff_ = 4.2 ± 2.06%, *p* = 0.373). Moreover, DT accuracy at Separate Feedback locations was greater than in both Movement Other (*M*_diff_ = 6.3 ± 1.42%, *p* = 0.004), and Irrelevant locations, (*M*_diff_ = 13.3% ± 2.39%, *p* = .001). This demonstrates that participants allocated attention simultaneously and to a similar extent to both the reaching target for action preparation and to the Separate Feedback locations for monitoring their movement accuracy.

To check whether there was an overall (main effect) or selective (interaction) enhancement of DT accuracy associated with the feedback and reaching target being in the same location, we conducted a 2 (Feedback Location: Same vs. Different) × 3 (DT Location: Movement vs. Movement Other vs. Irrelevant) repeated-measures ANOVA. In the Different condition, movement outcome accuracy information was presented opposite the reaching target; however, as no equivalent trials were conducted for the Same condition, this condition was not included in this analysis. As expected, this analysis confirmed a significant effect of DT-location *F*(2, 26), = 56.9, *p* < 0.001, η_*p*_^2^ = 0.891, with DT performance being highest at the Movement locations (*M* = 79.5 ± 2.4%), and lowest at the Irrelevant locations (*M* = 61.7 ± 2.1%), with Movement Other location falling in-between (*M* = 70.1 ± 2.7%), (all pairwise comparisons *p* ≤ 0.001). Importantly, however, Feedback Location had no significant main effect, *p* = 0.165, η_*p*_^2^ = 0.143, on the overall accuracy and there was also no interaction effect, *p* = 0.308, η_*p*_^2^ = 0.087. In short, where feedback was presented, whether at Movement locations or at a separate location, had no overall impact on DT accuracy in other locations. Therefore, participants allocated additional attention to the feedback locations in the Different condition to monitor their movement outcomes without significantly reducing the level of attention allocated to the Movement locations.

### Movement characteristics

We examined three movement parameters (RT, MT and endpoint accuracy). Note that in the following analyses Movement Other includes all Separate Feedback trials from the Different condition. We merged the data because pre-analyses (conducted as paired-samples *t* tests) showed that none of the tested movement parameters differed between the Movement Other and the Separate Feedback trials in this condition, all *p* > 0.40. By combining the data, we obtained an equal number of Movement Other trials for both Same and Different conditions. For all three measures, we will report the results of a 2 (Feedback Location: Same vs. Different) × 3 (DT Location: Movement locations, Movement Other locations, Irrelevant locations) repeated-measures ANOVA. Finally, as pre-analysis also confirmed that none of the movement parameters differed depending on whether or not the DT was accurately identified in a trial, all *p* ≥ 0.05, data were merged for all trials independent of discrimination accuracy.

#### Reaction time

**Fig. 4:**
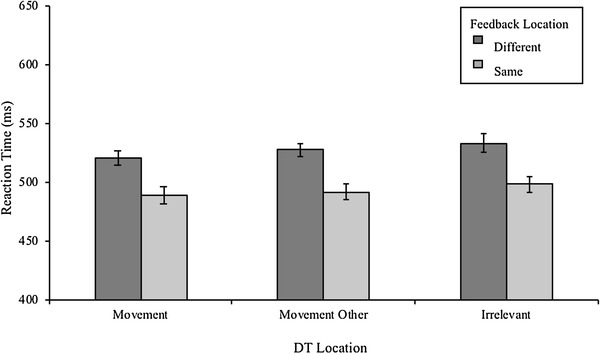
Reaction Time across conditions. DT Location refers to the location the discrimination target was presented. Error bars represent within-subjects standard error

As can be seen in Fig. 4, RTs were consistently faster when attention was not divided between feedback and movement locations, i.e. when feedback was presented in the same location as the action target.

Confirming this observation, the 2 × 3 repeated-measures ANOVA showed a significant main effect of Feedback Location, *F*(1, 13) = 7.43, *p* = 0.017, η_*p*_^2^ = 0.364. Interestingly, when movement outcome information was presented at the Movement targets, participants initiated their reaching movements, on average, 34 ms (± 12 ms) faster compared to when feedback was presented at a separate location opposite the reaching target. Allocating attention to monitor movement outcomes at a location separate to the Movement locations, therefore, seemed to negatively impact reaction times by delaying movement initiation. Moreover, we also found a significant main effect of DT Location, *F*(1, 13) = 5.4, *ε* = 0.61, *p* = 0.029, η_*p*_^2^ = 0.293. Descriptively, reaction times were fastest when the DT was presented at the Movement locations. Across both Same and Different conditions, participants initiated their reaching movements on average 11 ms (± 4 ms) faster in the Movement trials compared to the Irrelevant trials and 5 ms (± 2 ms) faster compared to the Movement Other trials. However, none of the post hoc comparisons were significant once corrected for multiple testing (all *p* > 0.08). No interaction effect between Feedback Location and DT Location was found, *p* = 0.405, η_*p*_^2^ = 0.067.

#### Movement time

**Fig. 5:**
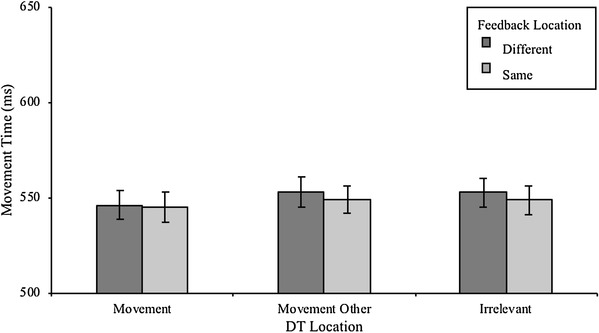
Movement Time across conditions. DT Location refers to the location the discrimination target was presented. Error bars represent within-subjects standard error

The same analysis as for RT was also applied to Movement Time (MT, see Fig. 5). Analysis revealed a main effect of DT Location on MT, *F*(2, 26) = 8.1, *p* = 0.002, η_*p*_^2^ = 0.563, but no main effect of Feedback Location, *p* = 0.843, η_*p*_^2^ = 0.003, and no interaction effect, *p* = 0.740, η_*p*_^2^ = 0.036. Participants executed the quickest movements when the DT was presented in the Movement locations. Pairwise comparisons show that reaching movements were about 6 ms (± 1 ms) longer in the Movement Other trials, *p* = 0.004, and 5 ms (± 2 ms) longer in the Irrelevant trials, *p* = 0.041, compared to the Movement trials. Movement times did not differ between the Movement Other and the Irrelevant trials, *p* = 1.00.

#### Endpoint accuracy

**Fig. 6:**
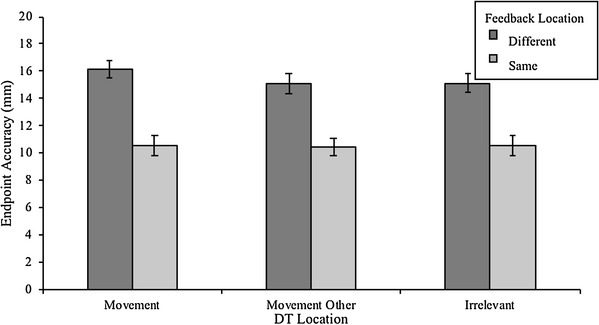
Endpoint accuracy refers to the 2D-distance of the finger’s final landing position in relation to the reaching target position. DT Location refers to the location where the discrimination target is presented. Error bars represent within-subjects standard error

Endpoint Accuracy, indicating the absolute Euclidean distance of the finger’s landing position from the target, for each DT Location in both Different and Same conditions, can be seen in Fig. 6. The figure illustrates that movements were considerably more accurate when the feedback and action locations coincided. As expected, the ANOVA revealed a main effect of Feedback Location, *F*(1, 13) = 12.93, *p* = 0.003, η_*p*_^2^ = 0.499. When feedback was presented at the *Movement* locations, participants were about 4.9 mm (± 1.4 mm) more accurate in their final landing position compared to the condition in which feedback was provided at a separate location. There was no main effect of DT Location on Movement Accuracy, *p* = 0.06, η_*p*_^2^ = 0.229, as well as no interaction effect, *p* = 0.061, η_*p*_^2^ = 0.228.

In conclusion, the perceptual results showed that in the Different condition, participants allocated attention to monitor movement accuracy feedback, even though it was provided at a spatially separate location to the reaching targets. The endpoint accuracy results suggest that participants attended separate feedback locations, even though dividing attention severely impacted their movement accuracy. Also, the movement accuracy impairment found in the Different condition occurred even though the amount of attention allocated to the Movement locations did not differ from Same condition.

## Discussion

In the current study, we investigated whether attention is directed to action targets solely for the purpose of action selection, or if attention is also directed predictively to action targets to monitor feedback about movement outcomes. Similar to previous research, we found that perceptual performance was enhanced at movement locations compared to movement-irrelevant locations, demonstrating a coupling between attention and action selection (e.g. Baldauf, Wolf, & Deubel, 2006; Baldauf & Deubel, 2008a, b; Deubel & Schneider, 1996; Deubel, Schneider & Paprotta, 1998; Kowler, Anderson, Dosher & Blaser, 1995). Crucially, however, when feedback about movement outcome accuracy was provided at a separate location from the reaching target, attention was equally allocated to both the Movement locations and the Separate Feedback locations. This means that during the preparation of the reaching movement, attention was already allocated to monitor the outcome of the intended action. Furthermore, while attention was divided across feedback and movement locations with no cost to overall discrimination accuracy, reaching movements were initiated faster and were more accurate when the feedback arose from the same location as the reaching target. Due to the difference in perceptual and reaching performance, we will discuss these results separately.

### Perceptual performance

Presenting movement accuracy feedback at Movement locations did not improve perceptual performance at this location compared to when feedback was provided at a separate location, showing that there is no additive effect of presenting visual feedback at the reaching goal. The persistent perceptual enhancement at the movement target, even in conditions in which these positions provide no information about movement outcome, appears consistent with an obligatory coupling of attention with action preparation and the notion that attention is required for action selection (Baldauf, Wolf, & Deubel, 2006; Baldauf & Deubel, 2008a, b; Deubel & Schneider, 1996; Deubel, Schneider & Paprotta, 1998; Kowler et al., 1995). However, it is important to consider that feedback about movement accuracy arises from the movement target in most natural situations. Thus, it may be possible that the allocation of attention to this location is overlearned or reflexive and cannot be easily withheld when it is not needed, since it almost always is. Kunde et al. (2018), who similarly found attention-for-feedback (or “effect-monitoring” as they named it), suggested that attention is partially allocated to task locations to monitor task effects and that delaying this feedback will result in attention remaining at this location. In line with Kunde and colleagues, our participants may have continued to attend the movement location to monitor their movement outcome effects, even when this information was provided elsewhere. While Kunde et al. (2018) did not spatially separate visual feedback from the task location, they did find that delaying the presentation of feedback delayed a second subsequent task, suggesting a strong relationship between attention, feedback and movements.

Interestingly, while perceptual performance at Movement locations remained consistent regardless of where feedback was provided, perceptual performance at the *Separate Feedback* locations did not differ from Movement locations. Although this suggests that attention can be divided across Movement and Separate Feedback locations without cost, it is important to take the movement characteristics into account, which suggest that movements are planned and executed less efficiently when attention is allocated to a feedback location that is spatially separated from the movement location (see “Reaching Performance” section for more detail). This observation is in line with findings from eye-movement studies in which separating perceptual and motor tasks was found to negatively impact the speed and precision of saccade execution (e.g., Born, Mottet, & Kerzel, 2014; Kowler et al., 1995; Moehler & Fiehler, 2014, 2015).

Furthermore, in the current study, the discrimination target was shown before the movement was initiated, and feedback was presented immediately after the movement was completed. It is known that attention allocation in hand movements can change over the course of the movement. Specifically, attention can be withdrawn from the target location within the first 300 ms (e.g., Deubel & Schneider, 2003). While attention for action selection is crucial for action initiation, attending the action outcome is relevant during or after the movement has been completed. Therefore, it is possible that the timing of the DT onset in our study tended to coincide with attention to the movement target. We would hypothesize that presenting the DT later in the movement planning process may result in a smaller attentional advantage at the movement target and a larger advantage at the feedback location. Further studies that vary the presentation time of the DT target relative to movement onset are required to gain more insight on the time course of attention allocation to the action target and the expected source of feedback.

A plausible alternative (or additional) reason for enhanced discrimination accuracy at the movement target and feedback locations is that participants may have prepared more than one action (Smith & Schenk, 2012), even if a movement is not ultimately executed to these locations. For example, while immediately planned action locations and source of immediate expected feedback locations may be a priority for the attentional system, possible movement targets from previous trials, but not in the current trial, may also receive more attention compared to locations that are never relevant to action. This is an important point, which we have tentatively addressed by including a comparison of these locations relative to both “Movement Other” locations (i.e., locations that could contain movement targets and feedback during the experiment but happen not to be relevant on the current trial) as well as to “Irrelevant” locations, which were never the target of an action nor a source of feedback. We found that locations that are possible movement targets in previous trials but not in the current trial (i.e. Movement Other trials) received more attention compared to locations that were never relevant to action. This means that our participants may have indeed prepared more than one action (Smith & Schenk, 2012). These results are broadly consistent with those of Van der Stigchel and Theeuwes (2005), who reported attentional facilitation for multiple saccade goals, even when participants are only required to execute a saccade towards one of them. Importantly, however, discrimination performance was enhanced at the current movement location relative to both of these baselines (i.e. Movement Other and Irrelevant), suggesting the immediate planning and execution of the action on that trial contributed to the enhanced discrimination performance we observed there.

An important consideration for our study is the potential effect that rewarding movement accuracy might have had on our findings. In a growing literature on the relationship between attention and reward, stimuli associated with rewards have been shown to draw attention (e.g. Anderson, 2016; Hickey, Kaiser & Peelen, 2015; McCoy & Theeuwes, 2016). The primary aim of these studies was to shed light on the role of reinforcement learning in determining how stimuli compete for our attention. However, in contrast to our study, in these previous studies, the “rewarded” stimulus was defined as the stimulus that elicited the response that led to the reward. Applying this definition to our experiment, the “rewarded” location would be the reaching target, rather than the feedback location, because the reward was attained by reaching accurately to the target. Attention seems to also prioritize locations that provide information about rewards (e.g. Bromberg-Martin & Hikosaka, 2009; Gottlieb, 2012), but this has not been as thoroughly investigated in human participants. The relationship between attention to reward and the perceptual benefits seen at the feedback locations may be an important factor in our experiment, and future studies are needed to explore the relative roles of financial reward and motor feedback in our results in more detail. More generally, however, even if our pattern of results depends on the presence of financial rewards, it remains an important result that attention was allocated predictively to expected locations of movement feedback (and/or reward information), and that it was also allocated to the movement target even though no new perceptual information about movement outcome could be expected to be gained by attending here.

Last, it is known that arrows can act as an automatic cue for attention, specifically as a symbolic cue with a strong (overlearned) spatial association (e.g. Tipples, 2002). In Online Resource 1, we present an additional experiment to disentangle the effect of the arrow alone from the effect of movement intention on attention allocation. Specifically, we examined whether the arrow cue could be responsible for enhancing perceptual performance at the movement target. The experiment was similar to Experiment 1 (Different condition), except that the arrow cue now pointed to the Separate Feedback target and not the Movement target in all trials and feedback was presented one position anti-clockwise from the Movement target instead of opposite to it. The perceptual results were essentially the same, with elevated accuracy at both the movement target and the feedback target relative to the other locations. Indeed, performance at the movement location in this supplementary experiment was numerically even slightly higher than in the experiment reported here (83 vs. 79%), though various differences between the two experiments make a direct comparison difficult. Nonetheless, these results suggest that the arrow cue alone does not elevate perceptual performance at the locations to which it points. These results are consistent with Baldauf, Wolf and Deubel (2006), who found no effect of arrows alone on perceptual discrimination performance in the absence of action selection. Although this contradicts previous results suggesting attention follows arrows automatically (e.g. Tipples, 2002), the experiments differ in a number of key aspects, including the number of possible target locations (2 vs. 8) and the measure of attention (discrimination accuracy vs. reaction time).

### Reaching performance

Although attention (as indicated by perceptual discrimination) was at a similar level at the Movement location when feedback was provided at a separate location, dividing attention between the movement and the feedback location slowed reaction times, and reduced the accuracy of the movement endpoints (see also Wirth et al. (2018), who separated visual and tactile feedback and found a negative impact on reaction times). At first glance, these findings may seem surprising, as it has been suggested that action control might not be constrained by the available (perceptual) attentional capacities (Enns & Liu, 2009; Liu, Chua, & Enns, 2008). Specifically, it has been previously argued that only movement planning, which relies on perceptual processing in the ventral stream, but not movement execution, which relies on visuomotor processing in the dorsal stream, shares attentional resources with perceptual tasks. In line with this prediction, Liu et al. (2008) showed that when participants were required to execute pointing movements concurrently with an additional perceptual task at a spatially separated location, reaction times were prolonged, while movement times and the accuracy of pointing movements (both indicating movement execution) were unaffected. The current experiment appears to contradict this finding as endpoint accuracy decreased when participants had to allocate attention to spatially separated feedback locations. This apparent contradiction might be accounted for by the fact that in Liu et al.’s experiment the perceptual target was presented longer in advance of the movement onset, such that processing resources only had to be shared at the very start of the trial. In contrast, in the current experiment, attention had to be allocated to the feedback position at the moment the finger reached the target (as feedback was displayed at the end of the movement), requiring participants to maintain attention on the feedback target throughout the trial and in particular toward the end of the movement. Hence, our findings contest the suggestion that perceptual tasks do not interfere with the online guidance of movements. Instead, the observed effects on movement characteristics suggest that simple actions such as reaching and pointing are indeed capacity limited and that perceptual and visuomotor processing share common processing resources (see also Kunde et al., 2007, Hesse & Deubel, 2011; Hesse, Schenk & Deubel, 2012). The fact that the dual task produced impaired motor performance but not impaired perceptual performance is consistent with the idea that predictive shifts of attention are primarily about specifying the parameters for the upcoming action, but less so with the notion that they are an involuntary consequence of motor programming per se (i.e. the data are consistent with VAM but not Premotor theory of attention).

Support for this interpretation also comes from a study by Similä and McIntosh (2015) in which participants were required to covertly monitor a flanker object during reaching. This resulted in a reduced ability to efficiently correct ongoing pointing movements, further suggesting that perceptual selection constrains the online guidance of hand movements. Similarly, it has been observed that the accuracy of grasping movements (as indicated by the early grip adjustment) suffers when participants are required to identify a perceptual target presented at a location different to a grasping target (Hesse & Deubel, 2011) or the contact positions of the fingers (Hesse, Schenk & Deubel, 2012). The current experiment shows that the same is true for reaching. This provides further support for the notion that there is a unitary control mechanism of visual attention that selects objects for perceptual processing and provides the spatial parameters required for an intended hand movement (Schneider, 1995).

## Conclusion

Previous research investigating the role of feedback in movement execution has focused on movement outcomes, mainly reaction and movement times, as a measure of attention allocation (for review see, Wulf, Shea, & Lewthwaite, 2010). This study is novel in using a perceptual task (discrimination) to directly examine the extent to which attention is allocated to expected augmented feedback locations prior to movement execution. In using this paradigm, we have bridged the gap between classic dual-task studies that show that attention and action are coupled (Baldauf, Wolf, & Deubel, 2006; Baldauf & Deubel, 2008a, b; Deubel & Schneider, 1996; Deubel, Schneider & Paprotta, 1998; Kowler et al., 1995), and motor learning research that shows, through movement outcomes, that attention is allocated to monitoring movement outcome feedback (see Wulf, 2007; Gray, 2011).

In conclusion, we confirmed in our study that attention is selectively allocated to reaching targets, even when no feedback about the movement outcome is available at this location. Moreover, if augmented terminal feedback about movement outcome is available in a location spatially separated from the reaching target, attention is allocated to monitor this location as well. While monitoring this location did not diminish perceptual performance at the reaching target in our study, dividing attention across separate reaching target and feedback locations increased movement initiation times and reduced the accuracy of movements. Future research may seek to further examine the role of the monetary reward provided in the current experiment, as well as the time course of attention allocation to action targets and feedback locations.

## Electronic supplementary material

Below is the link to the electronic supplementary material.

Supplementary material 1 (DOCX 4956 KB)
