# Supplementary material for: Shared attention for action selection and action monitoring in goal-directed reaching
Source: Psychol Res. 2018 Aug 10;84(2):313–26. doi: 10.1007/s00426-018-1064-x (PMC7040085; doi:10.1007/s00426-018-1064-x)
Supplement: Supplementary file 1 — Supplementary material 1 (DOCX 4956 KB) [file 426_2018_1064_MOESM1_ESM.docx]

**Shared attention for action selection and action monitoring in goal-directed reaching**

*Psychological Research*

Supplementary Material

Aoife Mahon^1&2^, Solveiga Bendžiūtė^1^, Constanze Hesse^1^, and Amelia R. Hunt^1^

^1^School of Psychology, University of Aberdeen, Aberdeen, UK

^2^Institute for Health Research, University of Bedfordshire, Luton, UK

Corresponding Author:

Aoife Mahon

Institute for Health Research

University of Bedfordshire, Luton, LU2 8LE

Email: aoife.mahon@beds.ac.uk

Tel: +44 (0)79 51770682

ORCID: 0000000311677693

**Supplementary Experiment:**

**Examining the influence of a central arrow cue on attention facilitation in reaching**

In our manuscript, we discussed the possible effect the central arrow cue may have had in orientating attention to reaching targets. Crucially, if arrows automatically direct attention to the location to which they are pointing (e.g. Tipples 2002), attention facilitation at reaching targets may be due to the central arrow that directs the movement, rather than (or in addition to) the processes involved in movement planning and execution. The studies conducted by Baldauf, Wolf, and Deubel (2006), on which our main experiment was based, had previously attempted to address this issue using a “discrimination-only” condition of the same task i.e. participants were asked to identify the discrimination target without performing any movements. They found no change in perceptual discrimination for the arrow alone, suggesting that facilitation at pointing and reaching targets is not due to the arrow cue, but to the movement the arrow directs.

Given that arrows have been previously shown to direct attention, however, we wanted to examine their effects in our experiment as well, to see if they could provide an alternative explanation for our results. The current experiment was similar to the Different condition in our main experiment, except the arrow cue now pointed to the *Separate Feedback* target, not the *Movement* target. Additionally, feedback was presented two positions anti-clockwise from the movement target instead of directly opposite. This design allowed a closer replication of the original studies by Baldauf and colleagues described above (2006), on which our hypothesis was based. Participants were required to execute goal-directed reaching movements while simultaneously identifying a discrimination target. If attention is indeed shifted automatically to the location the central arrow points to, discrimination performance should be enhanced more strongly at the separate feedback location than the reaching target in this experiment. Alternatively, if the movement execution is the main driver of attention, discrimination performance will continue to be enhanced at the reaching target.

**Method**

**Participants.** Thirteen undergraduate and postgraduate students of the University of Aberdeen (7 female, mean age range 22-40 years, average age of 24.2 years (7 females), were paid to participate. All participants had normal or corrected-to-normal vision and were right-handed by self-report. This research was conducted with the informed consent of each participant and was approved by the Psychology Research Ethics Committee, University of Aberdeen.

**Set-up.** The set-up used was identical to the main experiment.

**Stimuli and Procedure.** The stimuli and procedure were similar to the main experiment (Different Condition), except for the central arrow cue, the location of the feedback target and the way the feedback was provided. The central arrow, which previously indicated which location participants had to reach to, now cued the location from which participants would obtain feedback about their movement accuracy (see **Fig. S1**). Participants reached towards the target two positions clockwise from the cued feedback location. Immediately after completing their movement, participants received visual feedback regarding their movement accuracy. For an inaccurate movement the target (digital 8) changed red, while for an accurate movement it changed to green.

4. Masking

2. Feedback location cued

5. Feedback shown

3. DT and distractors shown

1. Start of trial

150 ms

SOA 50 ms, targets shown for 150ms

Cue accompanied by beep

Preview - 700 ms


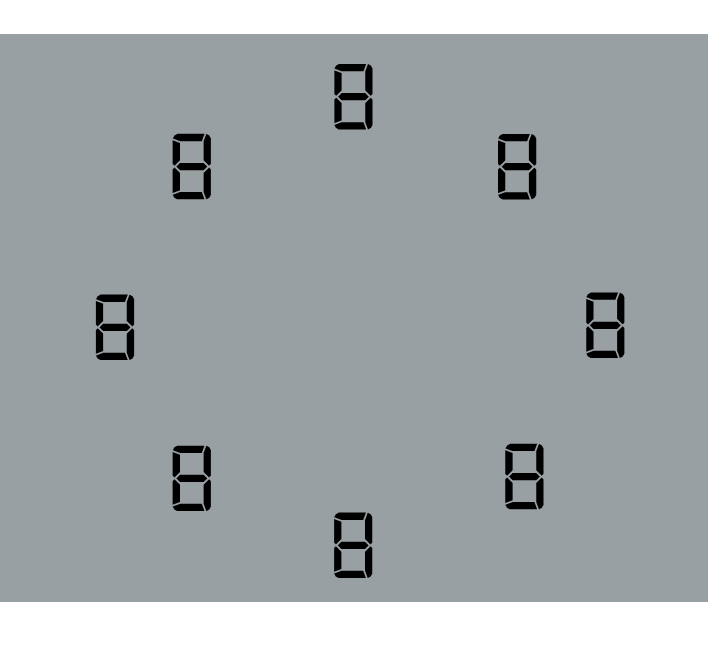

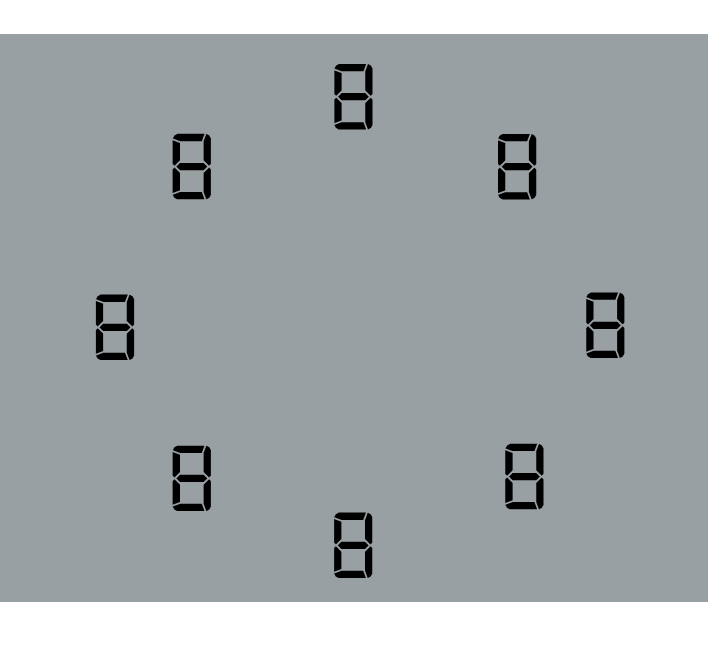

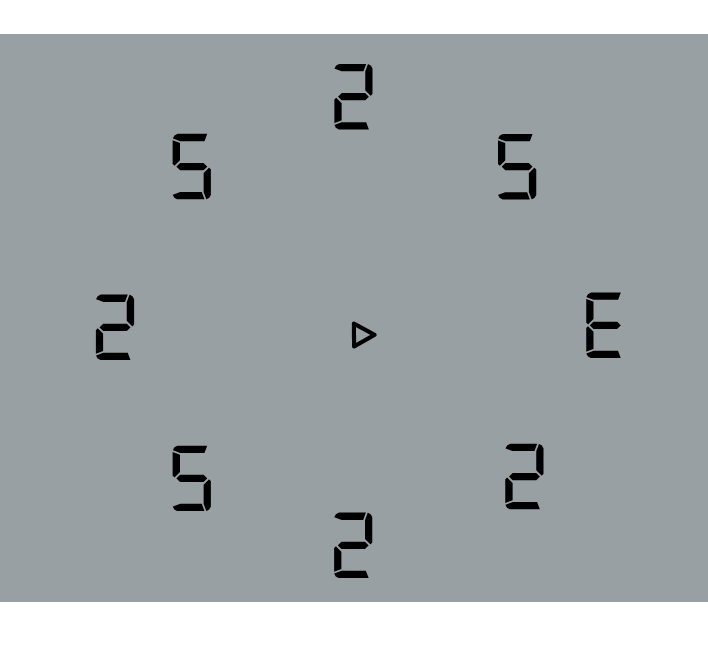

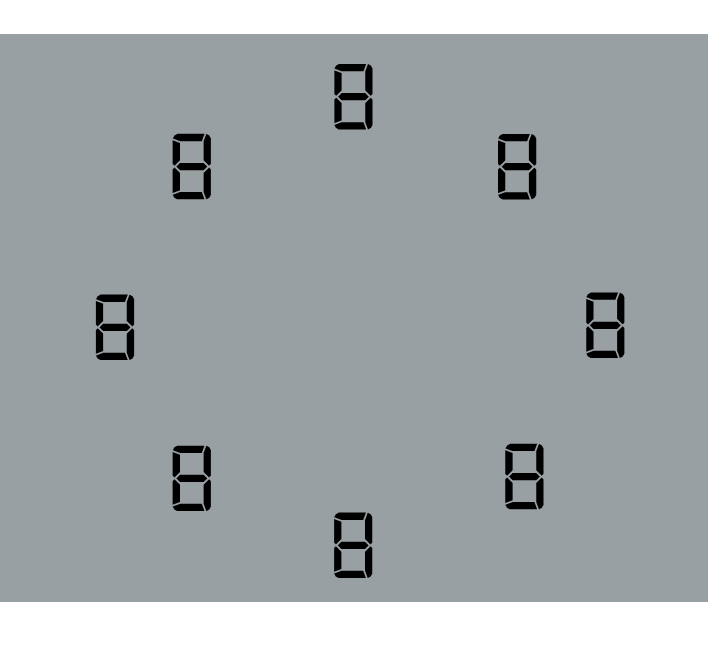

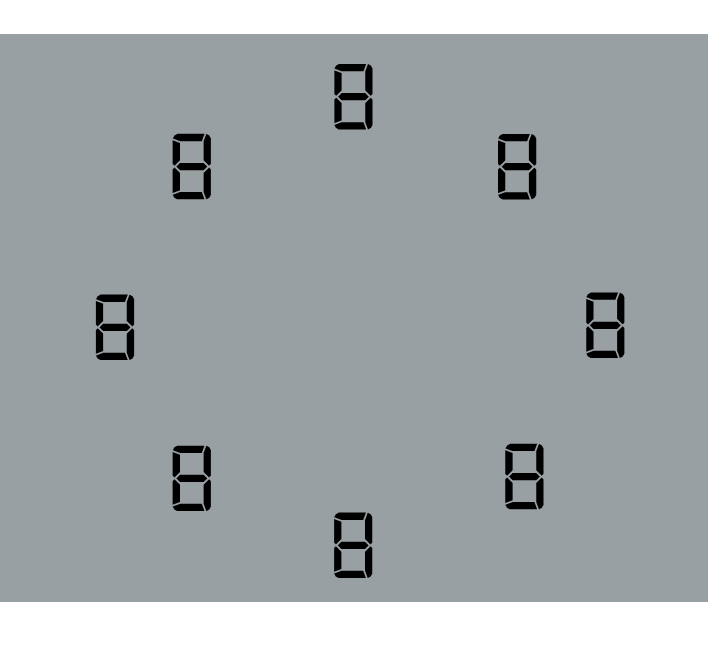


Participant points two

positions clockwise from cue


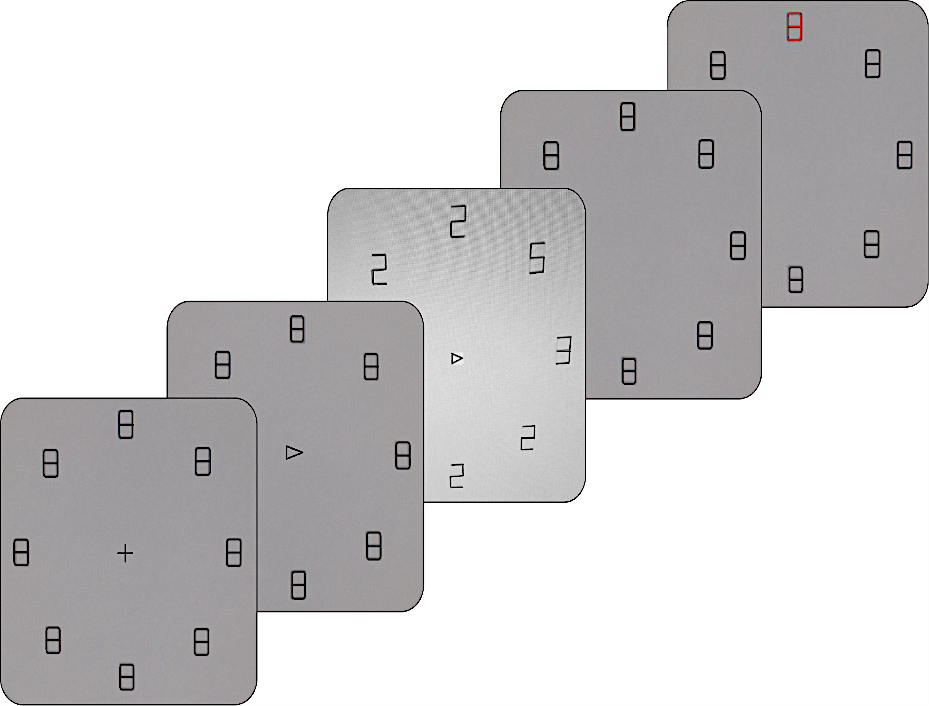

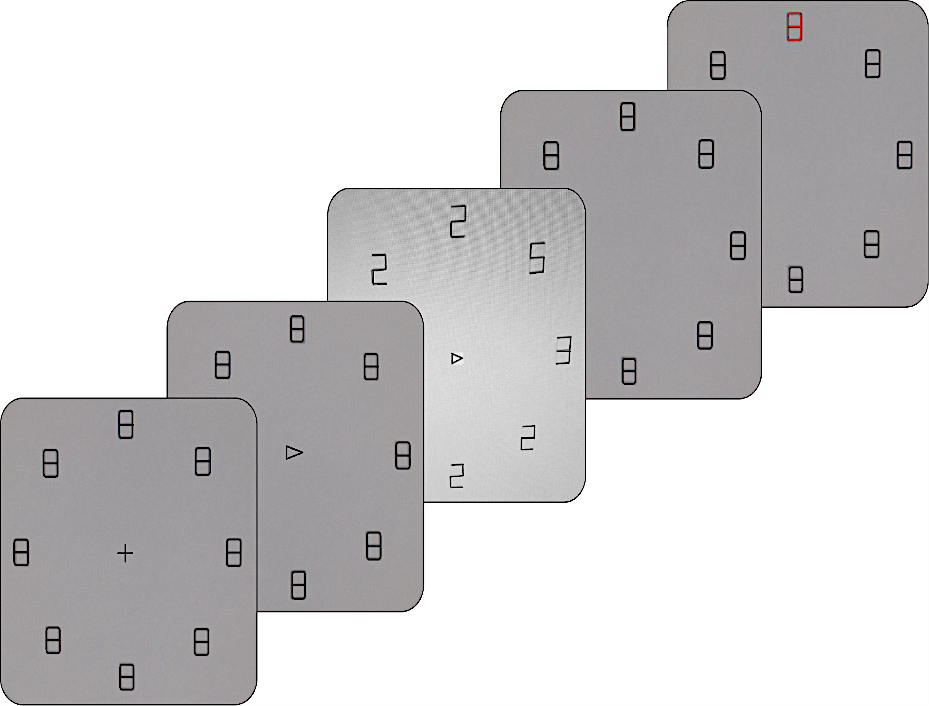


**Fig. S1** Sequence of stimuli. Feedback location was cued by the central arrow. The pointing target was always two positions clockwise from the feedback location.

**Design.** The main experimental manipulation was DT Location. DT Location was randomized within each block and fell into four categories:

- *Movement:* The DT was shown at the pointing target. Movement location was one of the four cardinal locations (i.e. “left”, “right”, “up” and “down”).
- *Separate Feedback*: The DT was shown at the feedback location (two positions anti-clockwise from the movement target and also one of the cardinal locations, see Figure S1 above).
- *Movement Other:* The DT was shown at a location that was a potential movement (and/or feedback) location on a previous trial (i.e. the four cardinal locations).
- *Irrelevant:* The DT was shown in one of the inter-cardinal locations that were never pointing locations i.e. a movement irrelevant location.

In total, each participant completed 480 trials over six blocks (80 trials per block), including one practice block (data not included in analysis) and five experimental blocks. Participants completed three blocks per session. Blocks lasted roughly 10-15 minutes. Each block consisted of 16 *Movement* trials, 16 *Separate* *Feedback* trials, 16 *Movement Other* trials, and 32 *Irrelevant* trials. Movement accuracy was only rewarded for experimental blocks (3 pence for each ‘accurate’ movement trial) resulting in a maximum possible additional reward of £12. No reward was given for inaccurate reaching movements. At the end of each block, participants were shown their monetary total for that block, as well as their overall earned total.

**Data analysis and rejection of trials.** Data rejection and analysis were equivalent to the main experiment. In total 45 trials (0.85%) were removed from further analysis due to reaction times being shorter than 200 ms.

**Results**

**Discrimination Target Accuracy**

Mean perceptual performance across all DT Locations can be seen in Figure S2. As can be seen, DT accuracy was highest at the *Movement* target, even when this location was no longer cued by the central arrow.


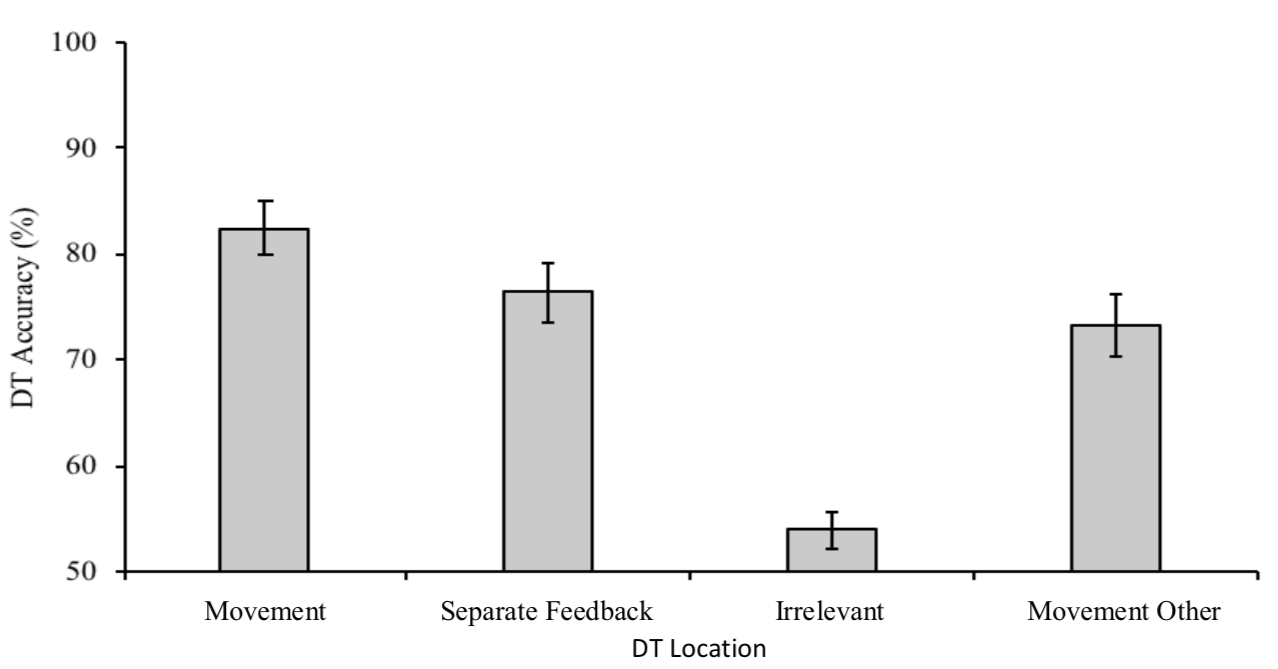


**Fig. S2** Bars represent mean discrimination target identification accuracy as a percentage. DT Location refers to the location where the discrimination target was presented. Chance is 50%. Error bars represent within-subjects standard error.

A one-way repeated measures ANOVA was conducted to compare the effect of DT Location on DT Accuracy. There was a significant effect of DT Location, *F*(3, 36) = 57.6, *p* < .001. Post hoc comparisons showed that perceptual performance was enhanced at *Movement* locations compared to both *Movement Other* locations (*M_diff_* = 9.2 ± 1.82%, *p* = .002), and *Irrelevant* locations (*M_diff_* = 28.5 ± 2.12%, *p* < .001). DT Accuracy at *Movement Other* location was enhanced compared to *Irrelevant* locations, (M_diff_=19.3 ± 1.59%, *p* < .001). DT identification accuracy at *Separate Feedback* locations was not significantly different from accuracy observed at the *Movement* locations*, (M_diff_= 6.1 ± 2.14%, p* = .086). DT accuracy at the *Separate* *Feedback* locations was enhanced compared to *Irrelevant* locations, (*M_diff_= 22.4 ± 2.68%, p* < .001), however, it did not differ significant from the *Movement Other* locations, (*M_diff_= 3.1 ± 3.09%, p>.99*).

## **Discussion**

The aim of the current experiment was to examine whether the central cue used in our experiment enhanced perceptual performance at movement targets by orientating attention to these locations, or if attention is allocated to movement targets because attention is needed for action selection. Descriptively, DT accuracy at the *Movement* location remained quite consistent between experiments (83% in main experiment vs. 79% in this experiment). This provides direct and clear evidence that attention facilitation at movement locations in this and similar dual-task studies is not due to these locations being cued by a central arrow (e.g., Baldauf, Wolf, & Deubel 2006; Deubel, & Schneider 1996).

Note, we did not find a difference in discrimination performance between the DT being presented at the separate feedback location as compared to other relevant locations. This may be due to the fact that feedback in this experiment was less informative than in our main experiment (i.e. participants were just informed whether their movement was accurate or not; but not about their exact final landing position). Hence, less attention may have been allocated to these location as participants could not gain valuable information about how to improve their performance in the next trial (i.e. overshoot vs. undershoot, further to the left or right etc.).

**References**

Baldauf, D., Wolf, M., & Deubel, H. (2006). Deployment of visual attention before sequences of goal-directed hand movements. *Vision Research*, *46*(26), 4355-4374.

Deubel, H., & Schneider, W. X. (1996). Saccade target selection and object recognition: Evidence for a common attentional mechanism. *Vision Research*, *36*(12), 1827-1837.

Tipples, J. (2002). Eye gaze is not unique: Automatic orienting in response to uninformative arrows. *Psychonomic Bulletin & Review*, 9(2), 314-318.
